# Plateaus and Afterglows: Theorizing the Afterlives of Gayborhoods as Post-Places

**DOI:** 10.1007/978-3-030-66073-4_16

**Published:** 2020-11-30

**Authors:** Jack Coffin

**Affiliations:** 17 Department of Architecture and Design, Alfred State University of New York, New York, USA; 18 Department of Urban and Regional Planning, University at Buffalo, Buffalo, NY USA; Fashion Marketing, University of Manchester, Manchester, UK

**Keywords:** Deleuze, Guattari, Place, Plateau, Afterglow, Assemblage, LGBTQ+

## Abstract

A number of commentators have acknowledged the decline of gayborhoods and the concomitant emergence of non-heteronormative diasporas in societies where sexual and gender diversity is normalized (Ghaziani 2015; Nash and Gorman-Murray 2017; Bitterman 2020). Academic studies tend to focus on the new lives that are being led beyond the gayborhood and the diminished distinctiveness of the territories left behind (e.g. Ghaziani 2014). In contrast, this chapter explores the possibility that gayborhoods can continue to influence sociospatial dynamics, even after their physical presence has diminished or disappeared altogether. Individuals and collectives may still be inspired by the memories, representations, and imaginaries previously provided by these erstwhile places. Indeed, the metaphor of a non-heteronormative diaspora relies on an ‘origin’ from which a cultural network has dispersed. In this sense gayborhoods can continue to function as post-places, as symbolic anchors of identity that operate even if they no longer exist in a material form, even if they are used simply as markers of ‘how far the diaspora has come’. The proposition that gayborhoods are becoming post-places could be more fully theorized in a number of ways, but the approach here is to adapt Deleuze and Guattari’s (1987: 22) notion of plateaus, which denote a “region of intensities whose development avoids any orientation towards a culmination point or external end”. From this perspective gayborhoods are not spatial phenomena that reach a climax of concentration and then disappear through dissipation. Instead, they can be described as becoming more intense and concrete in the latter half of the twentieth century before gradually fading after the new millennium as they disperse gradually into a diaspora as memories, habits, and so forth. Put another way, non-climactic gayborhoods leave ‘afterglows’, affects that continue to exert geographical effects in the present and near future. This conceptualization is consequential for theory, practice, and political activism, and ends the main body of this edited volume on a more ambitious note.

## Introduction

The sexualization of space, the spatialization of sexuality, and the relations of both to gender identities and gendered spaces; taken together, these phenomena represent a complex area of inquiry that has inspired decades of scholarship across academic disciplines.^1^ Looming large in these literatures are *gayborhoods*, concentrations of people, practices, and places that are associated with Lesbian, Gay, Bisexual, Trans*, Queer, and other non-heteronormative (LGBTQ +)^2^ cultures. Gayborhoods are typically dominated by commercial environments (such as bars) and events (like Pride festivals), often attracting the attention of mainstream brands (of which there are too many to enumerate), so it is unsurprising that marketing scholars and consumer researchers have taken an especial interest in these districts (see Coffin et al. 2016). However, recent discussions have focused on the decline, and even disappearance, of gayborhoods as LGBTQ + people no longer need spaces of (self-) segregation in an era of increasing societal acceptance (Ghaziani 2014, 2015) and deterritorializing technologies like dating applications for smartphones (Miles 2021). Yet, academic research suggests that the spatial diffusion of LGBTQ+ cultures away from gayborhoods does not signal a destructive de-spatialization but rather a more dynamic series of ongoing re-spatializations across a multitude of spaces (Bitterman 2020), many of which are peripatetic, ephemeral, and inconspicuous to varying degrees (Visconti 2008). It appears that the sociospatial dynamism of sexuality and gender appears to be in a “transitional stage toward a post-gay, post-binary-identity era” (Hess 2019: 230), in which the future of gayborhoods becomes more, rather than less, interesting for researchers of all kinds.

Many chapters in this edited volume have noted how gayborhoods have changed in the past and how they are continuing to change in the present, explicating their ever-changing role as places with personal and political significance. None of these chapters can provide a definitive prediction about the *future* of gayborhoods , not least because each is shaped by cultural, political, and infrastructural conditions that “are often unique to each place and must be investigated thoughtfully and carefully” (Bitterman 2020: 99). As so pertinently pointed out by Visser (2013: 269), “current Western theory is not only insufficient to explain gay spatial realities in the Western/Northern context itself, but it totally ignores (and is irrelevant to) the majority gay population located in very different and diverse settings elsewhere”. For example, Eeckhout et al. (2021) demonstrate how Antwerp, a typically North-Westerly cultural context, did not develop a gayborhood in the North American sense of the term. The conditions that constitute gayborhoods are also subject to change. For instance, the narrative of decline and disappearance is predicated on the assumption of widespread social liberalization, a trend which is well-evidenced but not inexorable (Pinker 2018). In recent years this historical trend has been challenged by the rise of populist politics and cultural xenophobia in the ostensibly ‘ post-gay’ developed world, as well as regressions elsewhere (Coffin et al. 2019). Acknowledging these contingencies, this chapter cannot aspire to a sweeping theorization of gayborhoods and their future; rather, I adopt the more humble ambition of providing an alternative line of thinking that may help scholars to sidestep the dualism of ‘ decline’ versus ‘endurance’ that implicitly underpins much of the existing literature (Coffin et al. 2016).

This alternative line of thinking is encapsulated in the concept of the ‘post-place’, which denotes how a disappearing gayborhood (or other physical locale) can continue to exert an influence, albeit an altered one, on the sociospatial dynamics of urban conurbations (and beyond). To help develop a more sophisticated conceptualization of post-places, I turn to the post-phenomenological perspectives provided by Gilles Deleuze and Félix Guattari, drawing particularly from their anti-climactic notions of plateaus and afterglows. Theorizing post-places may be useful for scholars interested in gayborhoods as a substantive topic area, but may also be transferred more generally to any instance where emplaced materiality gives way to more virtual and imagined forms of spatial affect, such as Hadrian’s Wall and other ruinous sites (e.g. Warnaby et al. 2010; Warnaby and Medway 2017).

## The Phenomenology of Place

Before developing my own Deleuzoguattarian lines of thinking, it is worthwhile outlining the phenomenological precepts of the place concept in order to preface the post-phenomenological positions that follow. The phenomenological point of departure is the importance of experience and the concomitant delineation between space and place (Cresswell 2004, 2013). From a phenomenological perspective *space* denotes meaningless material arrangements, while *place* describes meaningful manipulations of materiality into meaningful three-dimensional forms (Visconti et al. 2010). Stated succinctly, place is “a meaningful location” (Cresswell 2004: 7). Such phenomenological thinking has greatly influenced the study of gayborhoods and other LGBTQ + sites, albeit often implicitly. Sexuality and gender become salient when space becomes place, as meaningless environments become meaningful locations inflected by cultural connotations and sociosymbolic segregations. After years of research there persists a prevalent “notion that if space is not made gay or lesbian, then it *must* be straight”, such that “straight space [...] becomes the underlying frame with which we work: the space that gays subvert and the place that lesbians cohabit” (Bell et al. 1994: 32). Even when theorizations treat space as asexual and genderless, because it lacks humanity,^3^ they tend to be tacitly heteronormative in postulating that space is sexualized or gendered only by conspicuous material symbols and symbolic practices of LGBTQ+ identity (Kates 2002; Rosenbaum 2005; Visconti 2008).

Thanks to the phenomenological presuppositions, LGBTQ+ cultures have been informed by an underlying sociospatial dialectic of in/visibility, at least up until the turn of the millennium (Keating and McGloughlin 2005; Ghaziani 2015). The apparent decline and disappearance of gayborhoods in the years hence is partly explicable by the shift towards more subtle signals of sexuality (Brown 2006; Ghaziani 2014). However, this shift to subtlety also reflects a post-binary mood within non-normative cultures (Ghaziani 2011; Hess 2019), which arguably challenges the ocularcentric conceptualizations of conventional phenomenology and its heteronormative distinction between invisible straight-space and visible LGBTQ + places. Instead of conflating phenomenology with observable markers of identity, it may help to return to the origins of the term as a *philosophy of experience* (Thompson et al. 1989). As the links between visible symbolism and felt subjectivity dissolve, a particular place is defined via subjective experience, rather than through symbolically communicative objects or actions. This encourages scholars to adopt a more sensitive approach to theorizing the dynamics between sexuality, gender, space, and place. For instance, Ghaziani (2021) argues that urban scholars should listen more closely to LGBTQ + people, whose street-level experiences may facilitate a more nuanced understanding of gayborhoods than the ‘supra-individual’ patterns provided by demographers. In a similar vein, the minutiae of everyday experience may help to highlight how gayborhoods may become more than just physical places, as long as the phenomenology of experience is understood in a less conventional (but also more traditional) sense.

## Post-Phenomenological Perspectives

Despite the scholarly successes of phenomenological thinking, not all geographers or geographically-inclined academics presume phenomenological precepts. Many adopt different philosophical positions to advance alternative accounts of sociospatial phenomena, such as neo-Marxism (Soja 1980; Lefebvre 1991; Harvey 2005) or Thrift’s (2008) Non-Representational Theory (NRT), emphasizing how spatial arrangements can influence social phenomena without needing to be consciously recognized as places (Coffin and Chatzidakis 2021). Given that these alternative approaches de-centre the theoretical primacy of human experience, they can be grouped together under the heuristic heading of *post*-*phenomenology*, which retains the phenomenological interest in experience but jettisons the figure of “a fully formed subjectivity ‘in control’”, meaning that “experience is not individualized into ‘whole’ and coherent subjects, but rather presents a fractured sense of subjectivity” (Hietanen and Sihvonen 2020: 2). This term also encompasses the array of assemblage approaches that abound across social theory. The appellation of assemblage has many sources in social theory (Marcus and Saka 2006), but as a theoretical tool it was most extensively developed by Deleuze and Guattari (1983, 1986, 1987), becoming a way of conceptualizing “the world as constituted from more or less temporary amalgamations of heterogeneous material and semiotic elements, amongst which capacities and actions emerge not as properties of individual elements, but through the relationships established between them” (Canniford and Bajde 2016: 1). Accordingly, an assemblage approach can be used to conceptualize geographies as multi-scalar arrangements of relational activities (Anderson and McFarlane 2011; McFarlane and Anderson 2011; Allen 2011), or topologies (Cresswell 2013). As noted by Canniford et al. (2018: 235), “from an assemblage perspective, space is constructed from distributed entanglements […] in this view, spaces and the actions that occur there can be seen to be constructed from a broader network of things than initial appearances might warrant”. Nash and Gorman-Murray (2017) proposed that the concept of assemblage could enable scholars to account for the diverse and dynamic geographies of contemporary LGBTQ + cultures, but the application of this approach remains limited.

The assemblage is not the only concept that Deleuze and Guattari developed. As noted by Roffe (2016: 42–43), “further resources exist in their account that may yet be put to work”. Gilles Deleuze and Félix Guattari worked together on a number of texts including *Anti*-*Oedipus*, *Kafka: Towards a Minor Literature*, and *A Thousand Plateaus*. Much like Michel Foucault, their contemporary and Deleuze’s colleague, these authors are scholarly superstars who are often cited in passing by academics who have not necessarily read the original texts but rather engaged with them at a distance via secondary sources. This is perhaps partly due to their discursive style, which is “at times formidably difficult” (Roffe 2016: 42), “owing more to poetry than prose” (Coffin 2019: 2). Their writings draw from a range of sources and address a plethora of topics, adopting a non-linear structure that seeks to put their ‘ rhizomatic’ philosophy into practice. Yet a consistent theme in their work is an attempt to explain reality in terms of open-ended and open-to-change ‘becomings’ that blur and blend bodies of various kinds into machinic systems (Coffin 2019; Hietanen et al. 2019). Deleuze and Guattari constructed a cadre of concepts to crystalize their complex and changeable ontological outlook. Although the assemblage^4^ is one of the more famous of these concepts, the plateau represents another that may be productive for scholars interested in LGBTQ + topologies.

## Plateaus and Afterglows

Despite its eponymous status, the notion of the plateau is not developed extensively as a concept within the pages of *A Thousand Plateaus*. Rather it operates more as a performative device. In the ‘Author’s Note’ at the start of the book, Deleuze and Guattari (1988) explain that all of the chapters are ‘ plateaus’ that can be read in any order, except the conclusion which should be read last. Aside from this early exegesis, the plateau is only deployed a smattering of times within the remaining pages. However, Deleuze and Guattari are said to have supported imaginative interpretations of their work (Roffe 2016; Price and Epp 2016; Botez and Hietanen 2017), espousing what I have previously described as a “critical-creative spirit” (Coffin 2019: 8).^5^ When introducing his Deleuzoguattarian-inspired ‘ assemblage theory’, Manuel DeLanda (2006: 3) noted that “the relatively few places dedicated to assemblage theory in the work of Deleuze [ and Guattari]… hardly amount to a fully-fledged theory”. Instead, DeLanda (2006: 4) used his own conceptual creativity to draw these theoretical fragments together with resources from other sources, stating “readers who feel that the theory developed here is not strictly speaking Deleuze’s own are welcome to call it ‘ neo-assemblage theory’, ‘assemblage theory 2.0’, or some other name”. In a similar vein, I will draw inspiration from Deleuze and Guattari but define the nebulous notion of the plateau in my own terms, reworking it and the attendant concept of the afterglow into rhetorical resources apt for application to the study of erstwhile gayborhoods and other post-places.

**Fig. 16.1:**
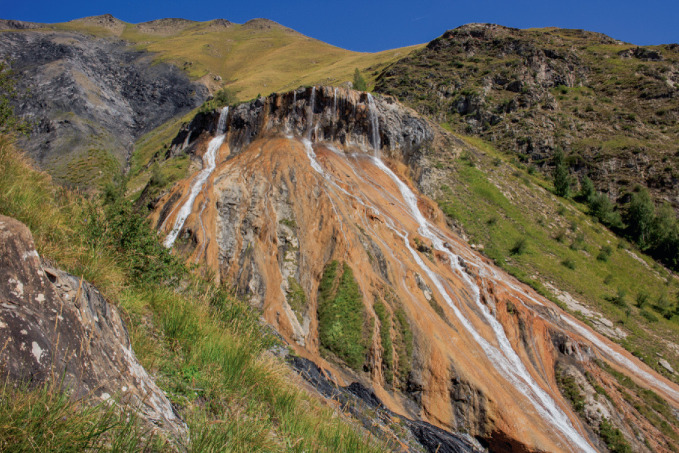
A geological plateau (*Source* Image by author)

 Deleuze and Guattari (1988: 21) describe how “ a plateau is always in the middle, not at the beginning or the end”. In terms of geology, a walker must ascend or descend along some other plane in order to reach the flat surface of a particular plateau. Metaphorically, this means that the term can imply changes in speed and orientation, as well as embeddedness within wider geographical and historical features (Fig. 16.1). They add an anthropological association when they argue that “Gregory Bateson uses the word ‘plateau’ to designate something very special: a continuous, self-vibrating region of intensities whose development avoids any orientation towards a culmination point or external end” ( Deleuze and Guattari, 1988: 21–22). Unlike the climax of a mountain peak or the nadir of the valley floor, a plateau is flat in all directions and does not culminate in anything except more of the same, unless one changes orientation and leaves the plateau via one of many sloped vectors. Referencing Bateson’s work with Balinese sexual culture, Deleuze and Guattari associate plateaus with intense experiences that do not result in orgasmic release but rather ongoing sensations. This contrasts sharply with the Western model of sexuality, as well as the Western model of thought and action generally. “It is a regrettable characteristic of the Western mind”, Deleuze and Guattari (1988: 22) write, “to relate expressions and actions to exterior or transcendent ends, instead of evaluating them on a plane of consistency on the basis of their intrinsic value”. In the broadest sense, then, plateaus refer to phenomena that have their own logics or arrangements, but are also embedded or interconnected into wider topologies, and thus affect the trajectories of those who pass across their planes.

The concept of the plateau can be applied to study places in ways that appreciate identity and relationality while avoiding linearity and teleology. The rise and decline of gayborhoods can be thought of as a linear narrative (Coffin et al. 2016), and one that emphasizes the goal-driven or ‘teleological’ activities of conscious place-makers. In contrast, Deleuzoguattarian thinking is open to the non-linear messiness of unexpected ruptures and connections by non-conscious forces that do not have any particular objective or orientation (Hietanen et al. 2019). It is post-phenomenological in the sense that it treats the experience of place as an ephemeral ‘becoming’ that emerges from the machinic entanglements of heterogeneous entities (Coffin 2019). Such becomings may be ephemeral, but their effects can be carried to other times and places in alternative forms, as when the direct experience of a place becomes a memory then applied to products and services (Brunk et al. 2018; Andéhn et al. 2019). Deleuze and Guattari (1988: 22) put this more poetically, writing that “lines leave one plateau and proceed to another like columns of tiny ants”. Thus, the place-as-plateau concept emphasizes the entanglement of geographical phenomena in ways that may not be foregrounded as prominently by the place-as-meaningful-location conceptualization (c.f. Cresswell 2004). Relationality is something that is often overlooked or left implicit in studies of spatiality (Coffin et al. 2016), except in cases where places are explicitly defined as ‘other’ (Foucault 1986; Chatzidakis et al. 2012; Roux et al. 2018), or when relationships to place are presented as proxies to interpersonal relationships (Debendetti et al. 2014; Rosenbaum et al. 2017). Drawing inspiration from Deleuze and Guattari, relational ontologies have become increasingly popular in geography (Massey 2005; Murdoch 2006) and marketing (Giovanardi and Lucarelli 2018; Canniford et al. 2018), representing a response to this theoretical tendency to elide sociospatial interconnectivity in favour of more isolated conceptualizations (Cresswell 2013). Plateaus build on this precedent.

**Fig. 16.2:**
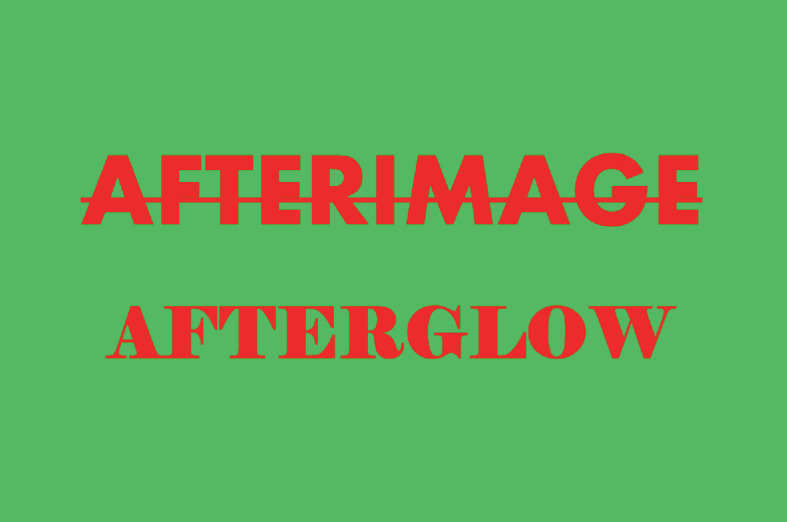
An example of a high contrast image that can create optical afterglows (*Source* Image by author)

The related notion of the afterglow does not come directly from Deleuze and Guattari but rather via Brian Massumi, a philosopher who also served as the English language translator of *A Thousand Plateaus.* In his translator’s foreword Massumi (1987, p.xiv) writes that “ in Deleuze and Guattari, a plateau is reached when circumstances combine to bring an activity to a pitch of intensity that is not automatically dissipated in a climax”. This description accords with the arguments made in the previous two paragraphs, but Massumi (1987, p.xiv) adds that “the heightening of energies is sustained long enough to leave an afterimage of its dynamism that can be reactivated or injected into other activities”. This is akin to the optical illusions that can be created by looking at a white surface after staring at certain brightly coloured images for several seconds (Fig. 16.2). Although this idea of an afterimage is only briefly mentioned, I feel it has much merit in helping to understand gayborhoods as *post*-places. It means that the relational effects of a place-plateau may linger, even if the physical location disappears, as long as the experience of that place is sufficiently intense to leave enduring marks, or ‘afterimages’. In turn, these afterimages allow a place to ‘live on’ as a post-place, moving from the visible marks considered by conventional phenomenological thought to the less visible, but indelible and influential, marks recognized by post-phenomenological thinking. As detailed below, the term ‘afterimage’ may be used to describe consciously recalled memories, but also preconscious habits, unconscious associations, and non-conscious environmental processes. Given that many of these phenomena are non-visual, I propose that the term *afterglow* as an alternative to the ocularcentric ‘afterimage’. Some glows may be visually apprehended but others keenly felt, and the term afterglow has associations apposite for scholars of sexuality and gender. Regardless of terminology, the crucial consequence of the afterimage or afterglow is identical—both terms refer to affective intensities whose energies are redirected into other forms and flows rather than released in a climatic cessation. It is this fading-out and fading-into that engenders the notion of a post-place, to which I presently turn.

## Post-Placing Gayborhoods

From a phenomenological perspective a gayborhood is an area of urban space that becomes meaningfully differentiated as an LGBTQ + place, with perceptions and practices (re)producing geographical arrangements within its perimeter that do not necessarily pertain elsewhere, at least not with as much potency. A gayborhood is born as it becomes distinctively LGBTQ+ and declines or ‘dies’ as its physical or imagined differences dissolve. However, from a post-phenomenological perspective a gayborhood can have an afterlife even if its physical presence is lost. This is because gayborhoods, like most meaningful places, produce intense affective experiences that leave their marks in the minds and bodies of humans, as well as in the heterogeneous bodies that constitute the non-human environment. Deleuze and Guattari (1987: 80) stress that “we may take the word ‘body’ in its broadest sense”. Bodies of water, political bodies, and bodies of knowledge all count in this broader definition of embodiment, as do the bodies of city streets and urban squares. If a plateau becomes sufficiently intense, as a physical place that can be experienced first-hand, then it can leave an afterglow that continues to exert an effect through the bodies of those that experienced this intensity. The plateau describes a place as a physical-sensual environment within a particular territory, while the afterglow denotes a post-place as an imaginary-symbolic effect that percolates through deterritorialized networks.

How might afterglows continue to affect bodies and perpetuate the afterlife of a post-place? At a conscious level, people may compare contemporary sites within an LGBTQ + diaspora to their memories of the erstwhile gayborhood. Stone (2021) shows that events like the Spanish Town Parade can link LGBTQ + people ‘vicariously’ to a sense of place and community, even one that no longer exists in a spatially concentrated form. In terms of plateaus and afterglows, this vicariousness may be analysed as a form of afterglow that percolates through parades and other events, indirectly linking a (post-) place to a wider diaspora. Even if a physical gayborhood persists, it may be influential more as an afterglow than as a physical place, and this afterglow may take on a greater importance in the urban fabric as the gayborhood evolves or dissolves. Interestingly, Stone’s account of the Spanish Town Parade highlights how heterosexual and even homophobic influences have begun to corrupt the contemporary manifestations of this non-normative event, thus suggesting that positively charged afterglows from the past may be diluted or distorted by competing influences in the present. Similarly, the identity of a place itself may become palimpsestic as the afterglows of previous incarnations compete with present-day interpretations and attempted re-appropriations in the near future. This topological layering of competing influences is vividly demonstrated in the case of Philadelphia (Niedt 2021), while the contribution by Podmore (2021) suggests that LGBTQ + places across a conurbation are defined by dynamic dis/identifications with a contested centrally located gayborhood.

A comparative analysis of gayborhoods with different historical and geographical trajectories, such as that conducted by Gorman-Murray and Nash (2020), suggests that while such places provide opportunities for community, belonging, and identity, they also constrain the emergence of other possible topologies. Thus, the decline of a gayborhood may actually be liberating for those subjectivities that have been largely occluded by the homonormative gayborhoods of the past (Frisch 2021), by rebalancing the centripetal and centrifugal forces within a particular milieu (Doan and Atalay 2021). If afterglows persist, then the limiting effects of gayborhoods may diminish without losing all of the positive effects that they used to provide. If so, it is also important for scholars to analyse instances where the afterglows of post-places fail to carry effects that were once engendered in the erstwhile physical place. Wienke et al. (2020) demonstrate how districts with a higher concentration of same-sex couples (i.e. gayborhoods) can reduce rates of depression and increase self-esteem. It is unlikely that such mental health benefits can be recreated by afterglows alone. Some, such as memories, may even make people feel *worse*, suggesting that afterglows may even reverse or invert the powers of a particular physical place. Then again, afterglows like habits may be able to carry across some of the positive affect from a place into a post-placed diaspora. Such speculations are best put to the empirical test.

Plateaus and afterglows add to a discursive repertoire which already abounds with metaphoric attempts to capture the ambulance, ambiguity, and ambivalence of contemporary LGBTQ + topologies. The notion of a diaspora, so eloquently employed by Bitterman (2020), makes little sense without a ‘home’ or central place from which a culture has dispersed. Analogously, the globally diffuse Jewish population would not be described as a diaspora without the notion of a physical place from which the Jewish culture emerged. The Jewish analogy also highlights how the idea of a homeland is not simply an academic reference point to define a diaspora but also an affectively-charged afterglow that lingers in the hearts and minds of deterritorialized cultures, even after many centuries. Such afterglows can also inspire action at the individual and collective level, as illustrated by the social movement to re-establish the Jewish homeland in the state of Israel. In the case of LGBTQ + diasporas, empirical research suggests that opinions may vary about whether the decline of gayborhoods should be lamented, celebrated, or some mixture of the two (Coffin et al. 2016; Ghaziani 2014), yet the identity of these diasporas are all affected by the afterimage of their lost local gayborhood. Subjects socialized into topologies that did not develop a clearly demarcated gayborhood—for example those in Italy (Visconti 2008) and Taiwan (Hsieh and Wu 2011)—may not ascribe to a diasporic logic.

**Fig. 16.3:**
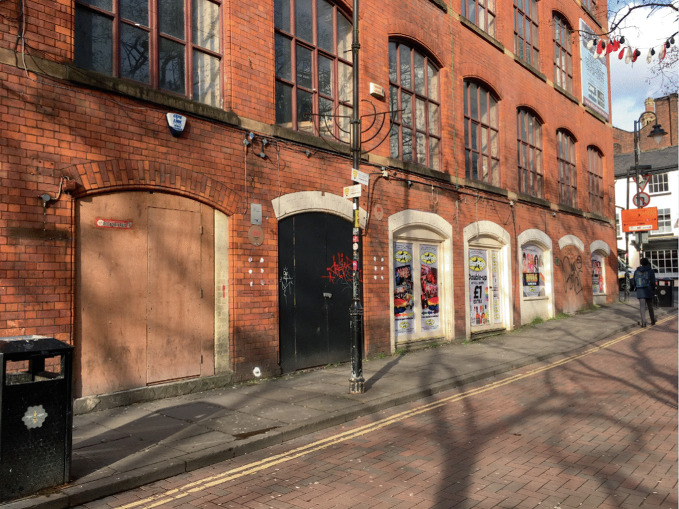
Closed premises in Manchester’s Gay Village (*Source* Image by author)

In contrast to the diaspora, Doan (2019: 5) provides a planetary metaphor, writing of gayborhoods as “mini suns around which LGBTQ individuals orbit, some closer, some further away”. What happens when the physical place disappears, no longer exerting its influence on individuals? One possibility is that gayborhoods which “burn too brightly may run out of fuel”, eventually exploding and then becoming ‘black holes’ (Doan 2019: 5). This might be one way of interpreting Manchester’s Gay Village, as a slow-motion supernova manifest in bankrupt LGBTQ + businesses and a black hole of boarded-up premises (Fig. 16.3). The slow diffusion designated by plateaus provides an alternative account to such climatic and cataclysmic conceptualizations, with gayborhood enterprises becoming untenable only because many other spaces become ‘friendly’ (Rosenbaum 2005) or ‘ post-gay’ (Brown 2006). In the case of Manchester it appears that the city center ‘gayborhood’ is less of a black hole and more of a white dwarf, diminishing physically but not quite losing its influence. Ghaziani (2014) has written about the physicality of disappearing gayborhoods remaining as historical markers, but the nuanced dynamics of how gayborhoods linger as afterglows could benefit from further analysis and theorization.

**Fig. 16.4:**
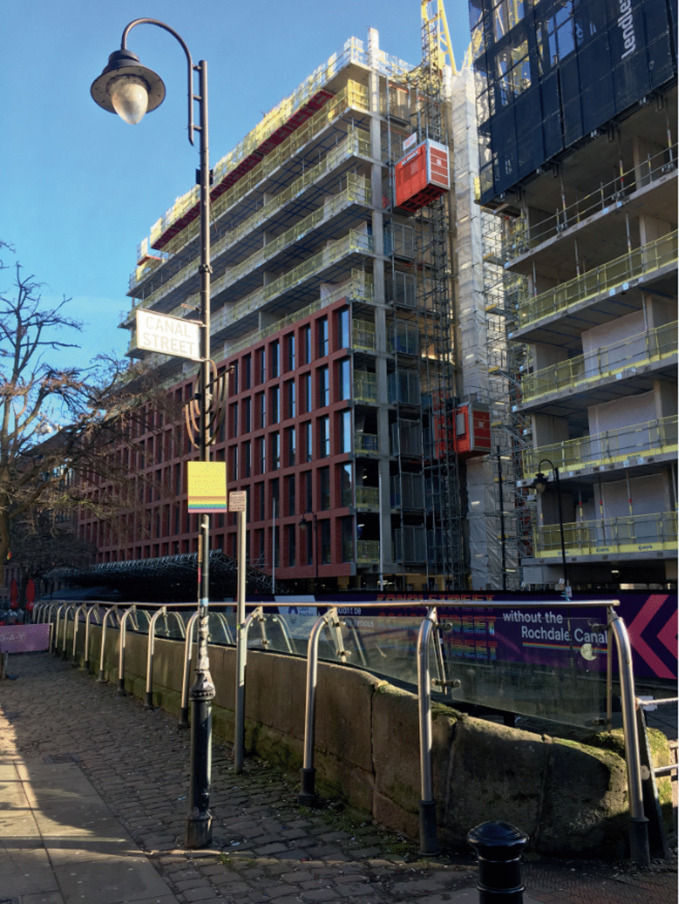
A new development in Manchester’s Gay Village (*Source* Image by author)

The plateau comes closest to the analogy of archipelagos proposed by Ghaziani (2019), especially as Deleuzoguattarian thinking redirects scholarly attention to influences “beneath the surface of salience” (Coffin 2019: 2), and thus considers the figurative landmass connecting islands beneath the waters. Put less figuratively, afterglows can also analyse and account for unconscious influences. A consciously perceived image in the individual mind or collective imagination may inspire action, but so too can an indelible and inarticulable mark left by an affective intensity. In marketing theory there has been a (re)turn to the psychoanalytic argument that some, if not most, of human subjectivity is unconsciously determined (Cluley and Desmond 2015). Such an appreciation might also be applied to afterglows, which may subtly shape sociospatial responses in ways that are difficult to pinpoint. In my own research many participants described Manchester’s Gay Village as an undesirable place in terms of atmosphere and service, yet they still felt a desire to visit and patronize this district for reasons that they could not articulate. This may necessitate a more psychoanalytic, or post-phenomenological, approach to analysis (Cluley and Desmond 2015; Hietanen and Sihvonen 2020). Posthuman sensibilities may also be advantageous, insofar as afterglows may also leave their marks in unconscious arrangements of non-humans. Geo-tagging photographs, writing customer reviews, or searching for a location on smartphone maps are all lingering records, even when physical places are lost. These may shape the results of internet searches and contribute to the ongoing performativity of marketing systems (Cluley and Brown 2015), which are increasingly automated in the post-marketing project of hyper-relevance (Darmody and Zwick 2020). These electronic afterglows may also affect the physical landscape by influencing urban planning, especially given that the prevailing penchant for ‘smart cities’ insists that all decisions should be based on big data rather than creative design (Fleming 2020). Here organic afterglows, such as the malleable memories of a place, may actually reduce resistance to capitalist logics of accumulation (Brunk et al. 2018), thus augmenting the electronic afterglows of data-driven urban development. As shown by Miles (2021), Grindr and other technologies create hybrid sociospatial arrangements that disrupt traditional, ‘low-tech’, theorizations of sexuality, gender, space, and place. Future research will need to carefully consider the complex configurations of human and non-human afterglows, and quickly; in Manchester’s Gay Village developments are already well underway (Fig. 16.4), and well on their way to erasing the gayborhood without much local resistance.

On the topic of future research, there are plenty of theoretical, practical, and political questions that invite further exploration. How affectively intense does a gayborhood (or other place) need to be in order to generate an afterglowing post-place? Do all places leave an afterglow, or is it only those that reach a certain threshold of affective intensity? If the latter, is there a typical threshold that affect needs to reach or does this vary greatly between contexts? Deleuze and Guattari (1987) write of singularities as events that lead to a qualitative change in an assemblage, a kind of ‘tipping point’ that is distinct from other changes. A simple example is that of heating water, with each incremental increase having little effect except that which tips over a ‘boiling point’ and transforms liquid water into steam (DeLanda and Harman 2017). In terms of places, it may be that only those that ‘boil over’ into spectacular experiences, powerful protests, or some other atypical threshold may leave afterglows. The Stonewall Inn Riots represent a case-in-point: the riots were a cultural watershed that set in motion many affects and actions, and the place is now a site of pilgrimage for many LGBTQ + people (see Ghaziani 2015). It is likely that the Stonewall will remain as a post-place even if the physical site is closed down, but other raided bars, which “failed to achieve the mythic status of Stonewall” (Armstrong and Crage 2006: 725), may not leave a lingering afterglow of affect.

Practical questions for non-academic stakeholders also abound. Should town planners, place marketers, and policy-makers attempt to anticipate post-places when designing places? Should they seek to nurture afterglows as valuable urban assets when gayborhoods and other once-significant spaces are displaced by gentrification? Or, are afterglows dangerous sources of attachment and resistance that should be avoided by those who wish capitalist accumulation and alteration to advance unabated? Place marketing studies of historical sites certainly suggest that afterglows can be valuable (Warnaby et al. 2010), but in vibrant places like cities afterglows may act as undesirable attachments that place branding consultants need to expunge (Warnaby and Medway 2013). Here one can observe how practical questions should provoke political considerations also. Who gets to decide which places should become post-places? Who benefits from afterglows and who loses out? Might afterglows become a post-rationalization for allowing important places to decline, presented as a salve for sociospatial refugees? For instance, selling cities as cosmopolitan and accepting may suggest that certain groups, such as trans* people, no longer need gayborhoods as safe spaces, especially in an era of digitalization. However, such narratives elide the enduring inequalities of access and assimilation (Coffin et al. 2016), and may be promoted by powerful actors intent on replacing LGBTQ + community spaces with more profitable real estate. More often than not, the political, practical, and theoretical effects of afterglows will form ambiguous admixtures that are difficult to disentangle.

## Conclusion: Beyond the Gayborhood in Space, Time, and Scholarship?

**Fig. 16.5:**
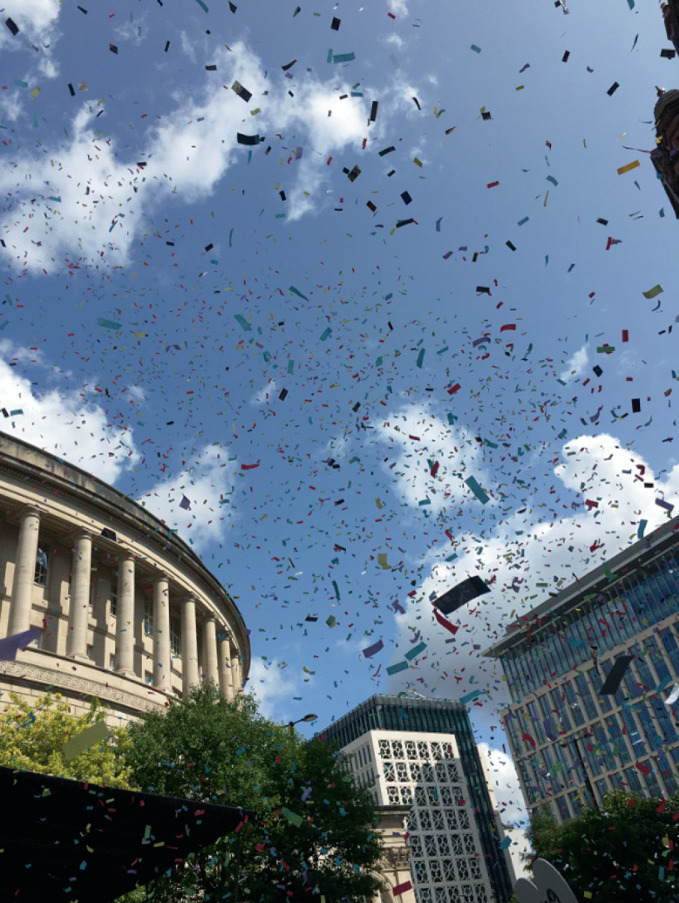
A photograph from the Manchaster Pride Parade (*Source* Image by author)

The notion of places as plateaus that leave post-phenomenological afterglows is one that extends the influence of a particular place beyond its geographical boundaries to a cultural diaspora that exists across wider urban, suburban, and rural geographies. This chapter applied such ideas to the case of gayborhoods, but they might also be appropriate when analysing a range of other (post-) places. These notions extend the power of place across space but also time, beyond the ostensible ‘death’ of a place as a physical site that can be visited and experienced in the phenomenological first-hand. While phenomenology focuses on the physical and representational aspects of places-as-environments, the post-phenomenological approach developed here may be applied also to places that are physically diminished, such as ruins (e.g. Warnaby et al. 2010; Warnaby and Medway 2017), or those that are replaced by less distinctive forms, such as the Powerscourt shopping mall in Dublin (Maclaran and Brown 2005). Such places live on in their afterglow, as memories, digital traces, and the collective imagination of culture. As plateaus and afterglows represent conceptual tools that can be transferred to other research areas, it might be argued that the emergence and evolution of LGBTQ + topologies should not be considered a discrete topic of interest for a niche group of scholars. Instead, we might be more ambitious and argue that gayborhoods are actually acute cases of broader processes that can be of general interest to geographers, sociologists, consumer researchers, and others. The lives and afterlives of gayborhoods are not simply stories of sexual and gender minorities, but a narrative structure that speaks to aspects of the universal human condition like acceptance, community, identity, change, and love (Fig. 16.5).^6^

## Footnotes

1 I hesitate to invoke the term ‘interdisciplinary’ here. Many authors develop their arguments within their own scholarly silos and most multi-author edited volumes anchor themselves within particular academic arenas (e.g. DeLamater and Plante 2015). I do not present this observation as a criticism—disciplinary depth can be just as valuable as anti-disciplinary aggregation. However, to my mind the present volume is perhaps one of the first to be truly *inter*-disciplinary in its ambition to assemble authors from all sorts of scholarly backgrounds.

2 Negotiations regarding the correct nomenclature for non-normative sexualities and genders are ongoing among academics and non-academics (Ghaziani 2011). In this chapter I follow Coffin et al. (2019) in adopting LGBTQ + as an acronym that carefully counterbalances succinctness and inclusiveness. However, I urge my contemporary readership to watch this space keenly. Future readers are likely to find LGBTQ + outmoded, just as using ‘gay’ as an overarching term seems somewhat awkward (to me, at least) at the present time.

3 Thus, this heteronormativity is also anthropocentric. Animals and plants have sexualized and gendered characteristics as well as territorial and spatial configurations. Is it such a stretch of the imagination to consider how these might also entwine? The vital materialism of Deleuze and Guattari, which builds on the earlier philosophical precedents of Spinoza and Nietzsche, posits that desires and (re)productive tendencies are found in everything from rocks to rainwater. Such philosophical positions could afford explorations of unfamiliar topologies that anthropocentric assumptions presently preclude (see Coffin, 2021).

4 It is worth noting here that the term assemblage is actually an Anglophone translation of the original French term *agencement*, and that the appropriateness of this translation is contested (Phillips 2006; Law 2009; Roffe 2016). Some, like Hietanen and Andéhn (2018: 552), prefer to use *agencement* as this is more suggestive of “the intertwined nature of all forces in such emergent events, rather than ‘ assemblage’, which connotes a more technical notion designating an arrangement of objects”.

5 The ‘critical’ is crucial here: it means thinking critically in the sense of questioning conventions and other taken-for-granted ways of thinking, feeling, acting, and becoming; however, it also means thinking critically of Deleuzoguattarian interpretations, and not treating Deleuze and Guattari’s licence of creativity as a kind of hyper-relativism masquerading as pragmatism (i.e. ‘my interpretation works within the context of this paper, so it cannot be criticised’). Buchanan (2015: 383) argues that Deleuze and Guattari wanted concepts to have “cutting edges”, so different interpretations of Deleuzoguattarian thinking can (and should) be evaluated.

6 This book chapter was written in 2019 and finished in the early months of 2020. In the weeks hence, the pathogen known as Covid-19 has proliferated to pandemic proportions. Although events are still unfolding, it can be said with some confidence that the concepts discussed in this chapter remain relevant. Afterglows abound under the aegis of quarantine culture, and many physical places may not survive the socioeconomic shocks that follow a period of local lockdown.

## References

[CR1] Allen J (2011). Powerful assemblage. Area.

[CR2] Andéhn M, Hietanen J, Lucarelli A (2019) Performing place promotion—on implaced identity in marketized geographies. Mark Theory, EarlyCite, pp. 1–22.

[CR3] Anderson B, McFarlane C (2011). Assemblage and geography. Area.

[CR4] Armstrong E, Crage S (2006). Movements and memory: the making of the Stonewall Myth. Am Sociol Rev.

[CR6] Bell D, Binnie J, Cream J, Valentine G (1994). All hyped up and no place to go. Gender, place and culture.

[CR8] Bitterman A (2020). Rainbow diaspora: the emerging renaissance of Gay neighbourhoods. Town Plann. Rev.

[CR9] Botez A, Hietanen J (2017). The rise of stagnancy and emergent possibilities for young radicals: Deleuze and the Perils of Idolatry. Ephemera.

[CR10] Brown G, Binnie J, Holloway J, Millington S, Young C (2006). Cosmopolitan camouflage: (Post-) Gay space in spitalfields. Cosmopolitan urbanism.

[CR11] Brunk K, Giesler M, Hartmann B (2018). Creating a consumable past: how memory making shapes marketization. J Cons Res.

[CR95] Buchanan I (2015) Assemblage theory and Its discontents. Deleuze Stud 9(3):382–392

[CR13] Canniford R, Bajde D (2016). Assembling consumption: researching actors, networks and markets.

[CR14] Canniford R, Riach K, Hill T (2018). Nosenography: how smell constitutes meaning, identity and temporal experience in spatial assemblages. Mark Theory.

[CR16] Chatzidakis A, Maclaran P, Bradshaw A (2012). Heterotopian space and the utopics of ethical and green consumption. J Mark Manag.

[CR18] Cluley R, Brown S (2015). The dividualised consumer: sketching the new mask of the consumer. J Mark Manag.

[CR19] Cluley R, Desmond J (2015). Why sychoanalysis now?. Mark Theory.

[CR20] Coffin J (2019) Deleuzoguattarian place marketing: becoming, between, beneath and beyond. J Place Manage and Dev, pp 1–20

[CR21] Coffin J (2021) “Posthuman Phenomenology: what are places like for Nonhumans?” In: Medway D, Warnaby, G Byrom J (eds) A research agenda for place branding. Edward Elgar Publishing, Cheltenham, UK

[CR89] Coffin J, Chatzidakis A (2021) The möbius strip of market spatiality: mobilizing transdisciplinary dialogues between CCT and the marketing mainstream. Acad Market Sci Rev. 10.1007/s13162-020-00191-8

[CR22] Coffin J, Banister E, Goatman A (2016). Revisiting the Ghetto: how the meanings of Gay districts are shaped by the meanings of the city. Adv Consum Res.

[CR92] Coffin J, Eichert C, Nolke A (2019) Towards (and Beyond) LGBTQ+ studies in marketing and consumer research. In: Dobscha S (ed) Handbook of research on gender and marketing. Edward Elgar Publishing, Cheltenham, UK, pp 273–293

[CR23] Cresswell T (2004). Place: a short introduction.

[CR24] Cresswell T (2013). Geographic thought: a critical introduction.

[CR25] Darmody A, Zwick D (2020). Manipulate to empower: hyper-relevance and the contradictions of marketing in the age of surveillance capitalism. Big Data & Soc.

[CR26] Debendetti A, Oppewal H, Arsel Z (2014). Place attachment in commercial settings: a gift economy perspective. J Cons Res.

[CR27] DeLanda M (2006). A new philosophy of society: assemblage theory and social complexity.

[CR28] DeLanda M, Harman G (2017). The rise of realism.

[CR29] Deleuze G, Guattari F (1983) Anti-oedipus, translated by Hurley R, Seem M, Lane H, Athlone, London

[CR30] Deleuze G, Guattari F (1986). Kafka: toward a minor literature, translated by Polan D, University of MN Press, Minneapolis, MN.

[CR31] Deleuze G, Guattari F (1987). A thousand plateaus: capitalism and schizophrenia.

[CR93] Deleuze G, Guattari F (1988) A thousand Plateaus, Trans. B. Massumi, Continuum, London, New York

[CR32] DeLamater J, Plante R (2015). Handbook of the sociology of sexualities.

[CR33] Doan P (2019). Cultural archipelagos or planetary systems. City & Community.

[CR34] Doan P, Atalay O, Bitterman A, Hess DB (2021). After the life of LGBTQ spaces: learning from Atlanta and Istanbul. The life and afterlife of Gay neighborhoods: renaissance and resurgence.

[CR35] Eeckhout B, Herreman B, Dhoest A, Bitterman A, Hess DB (2020). A Gay neighbourhood or merely a temporary cluster of “strange” bars? Gay bar culture in Antwerp. The life and afterlife of Gay neighborhoods: renaissance and resurgence.

[CR36] Fleming A (2020) The case for… making low-tech ‘dumb’ cities instead of ‘smart’ ones. The Guardian. Available at https://www.theguardian.com/cities/2020/jan/15/the-case-for-making-low-tech-dumb-cities-instead-of-smart-ones?utm_source=dlvr.it&utm_medium=twitter [Accessed on: 14/05/2020]

[CR37] Foucault M (1986). Of other spaces. Diacritics.

[CR39] Frisch M, Bitterman A, Hess DB (2021). A queer reading of the United States census. The life and afterlife of Gay neighborhoods: renaissance and resurgence.

[CR40] Ghaziani A (2011). Post-Gay collective identity construction. Soc Probl.

[CR41] Ghaziani A (2014). There goes the Gayborhood?.

[CR42] Ghaziani A, DeLamater J, Plante R (2015). The queer metropolis. Handbook of the sociology of sexualities.

[CR43] Ghaziani A (2019). Cultural archipelagos: new directions in the study of sexuality and space. City & Community.

[CR44] Ghaziani A, Bitterman A, Hess DB (2021). Why Gayborhoods matter: the street empirics of urban sexualities. The life and afterlife of Gay neighborhoods: renaissance and resurgence.

[CR45] Giovanardi M, Lucarelli A (2018). Sailing through marketing: a critical assessment of spatiality in marketing literature. J Bus Res.

[CR46] Gorman-Murray A, Nash CJ, Bitterman A, Hess DB (2020). Recovering the Gay village: a comparative historical geography of urban change and planning in Toronto and Sydney. The life and afterlife of Gay neighborhoods: renaissance and resurgence.

[CR90] Harvey D (2005) Space as a key word. In: Harvey D (ed) Spaces of neoliberalization: towards a theory of uneven geographical development. Franz Steiner Verlag, Stuttgart, pp 93–115

[CR47] Hess D (2019). Effects of gentrification and real-estate market escalation on Gay neighbourhoods. Town Plann Rev.

[CR48] Hietanen J, Andéhn M (2018). More than meets the eye: videography and production of desire in semiocapitalism. J Mark Manag.

[CR49] Hietanen J, Andéhn M, Wickström A (2019) The inhuman challenge: writing with dark desire. Organization, pp 1–13

[CR50] Hietanen J, Sihvonen A (2020) Catering to otherness: levinasian consumer ethics at restaurant day. J Bus Ethics. EarlyCite, pp 1–16

[CR51] Hill T, Canniford R, Mol J (2014). Non-representational marketing theory. Mark Theory.

[CR52] Hsieh M, Wu S (2011). Gay men’s identity attempt pathway and its implication on consumption. Psychol Mark.

[CR53] Kates S (2002). The protean quality of subcultural consumption: an ethnographic account of Gay consumers. J Cons Res.

[CR56] Keating A, McLoughlin D (2005). Understanding the emergence of markets: a social constructionist perspective on Gay economy. Consumption Markets & Culture.

[CR58] Law J, Turner BS (2009). Actor network theory and material semiotics. The new Blackwell companion to social theory.

[CR59] Lefebvre H (1991). The production of space.

[CR60] Maclaran P, Brown S (2005). The center cannot hold: consuming the Utopian marketplace. J Cons Res.

[CR91] Marcus GE, Saka E (2006) Assemblage. Theor Cult Soc 23(2–3):101–106. 10.1177/0263276406062573

[CR61] Massey D (2005). For space.

[CR62] Massumi B (1987) Translator’s foreword: pleasures of philosophy. In: Deleuze G, Guattari F (eds) A thousand plateaus. Continuum, London, UK

[CR63] McFarlane C, Anderson B (2011). Thinking with assemblage. Area.

[CR64] Miles S, Bitterman A, Hess DB (2021). Let’s (not) go outside: grindr, hybrid space, and digital queer neighbourhoods. The life and afterlife of Gay neighborhoods: renaissance and resurgence.

[CR65] Murdoch J (2006). Post-structuralist geography: a guide to relational space.

[CR66] Nash CJ, Gorman-Murray A (2017). Sexualities, subjectivities and urban spaces: a case for assemblage thinking. Gender, Place & Culture.

[CR68] Niedt G, Bitterman A, Hess DB (2021). A tale of three villages: contested discourses of place-making in central Philadelphia. The life and afterlife of Gay neighborhoods: renaissance and resurgence.

[CR70] Pinker S (2018). Enlightenment now: the case for reason, science, humanism, and progress.

[CR71] Podmore J, Bitterman A, Hess DB (2021). Far beyond the Gay village: LGBTQ urbanism and generation in montréal’s mile end. The life and afterlife of Gay neighborhoods: renaissance and resurgence.

[CR72] Price L, Epp A, Canniford R, Bajde D (2016). The heterogeneous and open-ended project of assembling family. Assembling consumption: researching actors, networks and markets.

[CR73] Rosenbaum M, Kelleher C, Friman M, Kristensson P, Scherer A (2017). Re-placing place in marketing: a resource-exchange place perspective. J Bus Res.

[CR74] Roux D, Guillard V, Blanchet V (2018). Of counter spaces of provisioning: reframing the sidewalk as a parasite heterotopia. Mark Theory.

[CR75] Phillips J (2006). Agencement/assemblage. Theory Culture Society.

[CR76] Roffe J, Canniford R, Bajde D (2016). The concept of the assemblage and the case of markets. Assembling consumption: researching actors, networks and markets.

[CR77] Rosenbaum M (2005). The symbolic servicescape: your kind is welcomed here. J Cons Behav.

[CR78] Soja E (1980). The socio-spatial dialectic. Ann Assoc Am Geogr.

[CR79] Stone AL, Bitterman A, Hess DB (2021). Wearing pink in Fairy Town: the heterosexualization of the Spanish Town neighborhood and Carnival Parade in Baton Rouge. The life and afterlife of Gay neighborhoods: renaissance and resurgence.

[CR80] Thompson C, Locander W, Pollio H (1989). Putting consumer experience back into consumer research: the philosophy and method of existential-phenomenology. J Cons Res.

[CR81] Thrift N (2008). Non-representational theory: space, politics.

[CR82] Visconti L (2008). Gays’ market and social behaviours in (de)constructing symbolic boundaries. Consumption, markets & culture.

[CR83] Visconti L, Sherry J, Borghini S, Anderson L (2010). Street art, sweet art? reclaiming the “public” in public place. J Cons Res.

[CR84] Visser G (2013). Challenging the gay Ghetto in South Africa: time to move On?. Geoforum.

[CR85] Warnaby G, Medway D, Bennison D (2010). Notions of materiality and linearity: the challenges of marketing the Hadrian’s Wall Place ‘product. Environ Plann A: Econ Space.

[CR86] Warnaby G, Medway D (2013). What about the ‘place’ in place marketing?. Mark Theory.

[CR88] Warnaby G, Medway D (2017) Pretty vacant? Implications of neglect and emptiness for urban aesthetics and place branding. In: Campelo A (ed) Handbook on place branding and marketing. Edward Elgar Publishing, Cheltenham, UK

[CR87] Weinke C, Whaley RB, Braatz R, Bitterman A, Hess DB (2020). Are “gay” and “queer-friendly” neighbourhoods healthy? assessing how areas with high densities of same-sex couples impact the mental health of sexual minority and majority young adults. The life and afterlife of Gay neighborhoods: renaissance and resurgence.

